# Disease characteristics and medication use in a multicenter cohort of children with juvenile idiopathic arthritis (JIA): preliminary analyses from the CARRAnet registry

**DOI:** 10.1186/1546-0096-10-S1-A46

**Published:** 2012-07-13

**Authors:** Sarah Ringold, Timothy Beukelman, Esi M Morgan DeWitt, Marc Natter, Peter A Nigrovic, Yukiko Kimura

**Affiliations:** 1 Children's Hospital Boston, Boston, MA, USA; 2 Cincinnati Children'sHospital, Cincinnati, OH, USA; 3 Hackensack University Medical Center, Hackensack, NJ, USA; 4 Stanford, CA, USA; 5 Seattle Children's Hospital, Seattle, WA, USA; 6 Boston, MA, USA; 7 University of Alabama-Birmingham, Birmingham, AL, USA

## Purpose

The CARRAnet Registry, a multicenter registry of children with rheumatic diseases in the U.S. organized by the Childhood Arthritis Rheumatology and Research Alliance (CARRA), began enrollment in May 2010. Our aims were to describe the characteristics of children with JIA enrolled into the registry to date and to identify characteristics associated with the use of biologic disease-modifying anti-rheumatic drugs (DMARDs).

## Methods

Data were extracted for all children with JIA who were enrolled up to December 28, 2010. Children who had ever received biologic DMARDs were compared to children who had not using relative risks (RR) and unpaired t-tests.

## Results

1072 children with JIA were enrolled during the first 7 months by 26 centers. The categorical characteristics of the cohort are shown in Table 1 and the continuous characteristics of the cohort are shown in Table 2. 77% had received at least one non-biologic DMARD at enrollment, most commonly methotrexate. 69% had received corticosteroids during their disease course, most frequently intra-articular (49%) and daily oral (36%). 45% of the cohort received one or more biologic DMARD during their disease course. The proportion of patients who received specific biologic agents is shown in Table 3. Children receiving biologic DMARDs were older at enrollment (mean age 13 years versus 10 years; p <0.001) and had a longer disease duration (mean 6 years versus 4 years; p<0.001). Children with imaging evidence of joint damage (RR 1.6; 95% CI: 1.3-1.9), positive RF (RR: 1.36; 95% CI 1.1 – 1.6), or positive anti-CCP (RR: 1.35; 1.1-1.7) were more likely to have received a biologic DMARD. Children with oligoarthritis were less likely to have received a biologic DMARD than other categories.

**Table 1:** 

Characteristuc	N (%)
*JIA Category*	
Systemic	87 (8)
Polyarticular (RF-)	334 (31)
Polyarticular (RF+)	266 (25)
Extended Oligoarticular	91 (9)
Psoriatic	56 (5)
Enthesitis-Related	110 (10)
Undifferentiated	37 (3)
Other or unknown	14 (1)
*Female*	783 (73)
*Race*	
White	965 (89)
Black or African American	62 (6)
Asian	29 (3)
Other	54 (5)
*Ethnicity*	
Hispanic or Latino	101 (9)

*Positic Serology*	
ANA	483 (45)
RF (initial)	88 (8)
RF (confirmatory)	46 (4)
Anti-CCP	51 (5)
HLA-B27	95 (9)

*Uveitis*	
Current	46 (4)
Past	74 (7)

*ACR Functional Class*	
Class I	872 (81)
Class II	179 (17)
Class III	28 (3)
Class IV	4 (<1)

*Health-related quality of life*	
Excellent	242 (23)
Very good	437 (41)
Good	352 (33)
Poor	28 (3)
Very Poor	1 (<1)
*Imaging evidence of joint damageI*	265 (25)

**Table 2:** 

Characteristic	Mean (Median)	Range
Age at enrollment (years)	11 (12)	<1 – 22

Age at symptom onset (years)	7 (5)	<1 – 16

Age at first rheumatology visit (years)	7 (7)	<1 – 21

Disease duration (years)	5 (4)	0 – 18

Duration between sympton onset and first rheumatology visit (years)	1 (<1)	0 – 12

Number of active joints	2 (0)	0 – 38
Physician global assessment of disease activity	2 (1)	0 – 9
Parent/patient assessment of disease acticity	2 (1)	0 - 10
Parent/patient assessment of overall well-being	2 (2)	0 – 9
Patrent/patient asessment of pain	3 (2)	0 – 10
CHAQ score	0.35 (0.125)	0 – 3

**Table 3:** 

Biologic medication	Current use	Prior use
	N (% of biologic users)	N (% of biologic users)

*TNF-alpha inhibitirs*		
Adalimumab	78 (7)	70 (6)
Cartolizumab	4 (<1)	0
Etanercept	214 (20)	183 (17)
Gollimumab	5 (<1)	4 (<1)
Inflximab	46 (4)	46 (4)
*IL-1 Inhibitors*		
Ankira	14 (1)	22 (2)
Rilonacept	2 (<1)	2 (<1)
*Other biologic agents*		
Abatacept	24 (2)	8 (<1)
Rituximab	2 (<1)	8 (<1)
Tocilizumab	6 (<1)	0 (<1)

## Conclusion

The majority of patients with JIA enrolled into the CARRAnet registry has relatively low disease activity, minimal disability, and have received at least one DMARD. Positive anti-CCP or RF, joint damage on imaging, older age at enrollment and longer disease duration were associated with biologic DMARD use. Limitations include the underrepresentation of non-English speaking families and enrollment bias. Continued enrollment into this cohort will support future analyses with increased sample sizes and the potential for longitudinal data analysis.

## Disclosure

Sarah Ringold: None; Timothy Beukelman: None; Esi M. Morgan DeWitt: None; Marc Natter: None; Peter A. Nigrovic: None; Yukiko Kimura: None; CARRAnet Investigators: None.

